# Role of *N*-Arachidonoyl-Serotonin (AA-5-HT) in Sleep-Wake Cycle Architecture, Sleep Homeostasis, and Neurotransmitters Regulation

**DOI:** 10.3389/fnmol.2017.00152

**Published:** 2017-05-30

**Authors:** Eric Murillo-Rodríguez, Vincenzo Di Marzo, Sergio Machado, Nuno B. Rocha, André B. Veras, Geraldo A. M. Neto, Henning Budde, Oscar Arias-Carrión, Gloria Arankowsky-Sandoval

**Affiliations:** ^1^Laboratorio de Neurociencias Moleculares e Integrativas, Escuela de Medicina, División Ciencias de la Salud, Universidad Anáhuac MayabMérida, Mexico; ^2^Grupo de Investigación en Envejecimiento, División Ciencias de la Salud, Universidad Anáhuac MayabMérida, Mexico; ^3^Grupo de Investigación Desarrollos Tecnológicos para la Salud, División de Ingeniería y Ciencias Exactas, Universidad Anáhuac MayabMérida, Mexico; ^4^ Intercontinental Neuroscience Research Group; ^5^Endocannabinoid Research Group, Istituto di Chimica Biomolecolare, Consiglio Nazionale delle RicerchePozzuoli, Italy; ^6^Laboratory of Panic and Respiration, Institute of Psychiatry, Federal University of Rio de JaneiroRio de Janeiro, Brazil; ^7^Postgraduate Program, Salgado de Oliveira UniversityRio de Janeiro, Brazil; ^8^Faculty of Health Sciences, Polytechnic Institute of PortoPorto, Portugal; ^9^Institute of Psychiatry, Federal University of Rio de JaneiroRio de Janeiro, Brazil; ^10^Dom Bosco Catholic UniversityRio de Janeiro, Brazil; ^11^Faculty of Human Sciences, Medical School HamburgHamburg, Germany; ^12^Physical Activity, Physical Education, Health and Sport Research Centre (PAPESH), Sports Science Department, School of Science and Engineering Reykjavik UniversityReykjavik, Iceland; ^13^Department of Health, Physical and Social Education, Lithuanian Sports UniversityKaunas, Lithuania; ^14^Unidad de Trastornos del Movimiento y Sueño (TMS), Hospital General “Dr. Manuel Gea González"Ciudad de México, Mexico; ^15^Centro de Investigaciones Regionales “Dr. Hideyo Noguchi”, Universidad Autónoma de YucatánMérida, Mexico

**Keywords:** sleep, dopamine, modafinil, cannabidiol, sleep deprivation

## Abstract

The endocannabinoid system comprises several molecular entities such as endogenous ligands [anandamide (AEA) and 2-arachidonoylglycerol (2-AG)], receptors (CB_1_ and CB_2_), enzymes such as [fatty acid amide hydrolase (FAHH) and monoacylglycerol lipase (MAGL)], as well as the anandamide membrane transporter. Although the role of this complex neurobiological system in the sleep–wake cycle modulation has been studied, the contribution of the blocker of FAAH/transient receptor potential cation channel subfamily V member 1 (TRPV1), *N*-arachidonoyl-serotonin (AA-5-HT) in sleep has not been investigated. Thus, in the present study, varying doses of AA-5-HT (5, 10, or 20 mg/Kg, i.p.) injected at the beginning of the lights-on period of rats, caused no statistical changes in sleep patterns. However, similar pharmacological treatment given to animals at the beginning of the dark period decreased wakefulness (W) and increased slow wave sleep (SWS) as well as rapid eye movement sleep (REMS). Power spectra analysis of states of vigilance showed that injection of AA-5-HT during the lights-off period diminished alpha spectrum across alertness in a dose-dependent fashion. In opposition, delta power spectra was enhanced as well as theta spectrum, during SWS and REMS, respectively. Moreover, the highest dose of AA-5-HT decreased wake-related contents of neurotransmitters such as dopamine (DA), norepinephrine (NE), epinephrine (EP), serotonin (5-HT) whereas the levels of adenosine (AD) were enhanced. In addition, the sleep-inducing properties of AA-5-HT were confirmed since this compound blocked the increase in W caused by stimulants such as cannabidiol (CBD) or modafinil (MOD) during the lights-on period. Additionally, administration of AA-5-HT also prevented the enhancement in contents of DA, NE, EP, 5-HT and AD after CBD of MOD injection. Lastly, the role of AA-5-HT in sleep homeostasis was tested in animals that received either CBD or MOD after total sleep deprivation (TSD). The injection of CBD or MOD increased alertness during sleep rebound period after TSD. However, AA-5-HT blocked this effect by allowing animals to display an enhancement in sleep across sleep rebound period. Overall, our findings provide evidence that AA-5-HT is an important modulator of sleep, sleep homeostasis and neurotransmitter contents.

## Introduction

The endocannabinoid system is a complex biological arrangement that exerts multiple modulatory functions (Kendall and Yudowski, 2016; Ligresti et al., 2016; Argueta and DiPatrizio, 2017; Bennett et al., 2017; Dos Anjos-Garcia et al., 2017; Sun et al., 2017). The components of the endocannabinoid system include the most studied endocannabinoids anandamide (AEA) and 2-arachidonoylglycerol (2-AG; Di Marzo and Piscitelli, 2015; Yuan et al., 2016; Biernacki and Skrzydlewska, 2016; Correa et al., 2016; Huang et al., 2016; Lu and Mackie, 2016; Fakhoury, 2017). The endocannabinoids bind to transmembrane proteins denominated CB_1_ and CB_2_ cannabinoid receptors (Kendall and Yudowski, 2016; Kruk-Slomka et al., 2016; Chen et al., 2017; Fitzpatrick and Downer, 2017; Mallipeddi et al., 2017). Additionally, it has been reported that the main enzymes responsible for the hydrolysis of AEA and 2-AG are fatty acid amide hydrolase (FAAH) or monoacylglycerol lipase (MAGL), respectively (Piomelli, 2014; Biernacki and Skrzydlewska, 2016; Janssen and van der Stelt, 2016). Lastly, several pieces of evidence have shown that anandamide membrane transporter (AMT) also displays a key role in modulating multiple biological functions (Leung et al., 2013; Nicolussi and Gertsch, 2015). Among the diverse findings that suggest the involvement of the endocannabinoid system in regulating several neurobiological phenomena, different reports have indicated that the sleep-wake cycle is likely under control of this system (Santucci et al., 1996; Murillo-Rodríguez et al., 1998, 2001, 2003, 2008a, 2011a, 2013, 2016; Herrera-Solis et al., 2010; Rueda-Orozco et al., 2010; Pava et al., 2014, 2016). Due to recent results regarding the relationship between AEA and the transient receptor potential cation channel subfamily V member 1 (TRPV1) also known as the capsaicin receptor or vanilloid receptor 1, increasing scientific interest is addressing the neuromolecular properties of TRPV1 (Tóth et al., 2009; Lowin and Straub, 2015; Chen et al., 2016; Kirkedal et al., 2016). An experimental approach aimed to describe the neuromolecular role of TRPV1 has included pharmacological means by using *N*-arachidonoylserotonin (AA-5-HT). For example, it has been reported that this drug is a dual blocker at FAAH/TRPV1 inducing anxiolytic-like effects in mice (Micale et al., 2009). Moreover, different doses of AA-5-HT (0–0.5 nmol) administered into the basolateral amygdala prior to elevated plus maze testing increased time spent into the open arms (John and Currie, 2012). In addition, it has been shown that AA-5-HT induces anticonvulsant effects (Micale et al., 2009; Vilela et al., 2014), reduces depression-like behavior (Navarria et al., 2014; Kirkedal et al., 2016) and promotes recovery from stress (Sartim et al., 2017). Despite this accumulating body of evidence regarding the neurobiological role of AA-5-HT, no evidence is available on whether this compound might modulate the sleep-wake cycle. Thus, we investigated the effects of AA-5-HT (5, 10, 20 mg/Kg, i.p.) on sleep as well as extracellular contents of neurotransmitters linked to the sleep-wake cycle such as dopamine (DA), norepinephrine (NE), epinephrine (EP), serotonin (5-HT), as well as adenosine (AD). Moreover, to provide further evidence regarding the putative sleep-inducing properties of AA-5-HT, we tested whether this drug would block the alertness caused by the phytocannabinoid cannabidiol (CBD, 30 mg/Kg, ip; Murillo-Rodríguez et al., 2006, 2008b, 2011b; Kim, 2012) or the stimulant drug modafinil (MOD, 30 mg/Kg, i.p.; Sukhal et al., 2015; Wang et al., 2015; Barateau et al., 2016). In addition, the role of AA-5-HT in sleep homeostasis was addressed in CBD- or MOD-treated animals that were sleep deprived. Our study provides novel findings about the possible role of AA-5-HT in sleep modulation.

## Materials and Methods

### Experiment 1: Effects on Sleep after Injections of AA-5-HT during Either the Lights-On or Lights-Off Period

#### Experimental Animals

Male Wistar rats (*n* = 50; 250–300 g) were singly housed in polycarbonate cages (48.26 cm × 26.67 cm × 20.32 cm; Harlan Laboratories. México) under light-dark cycle (lights-on: 07:00–19:00h), humidity controlled (60 ± 10%) and constant temperature (21 ± 1°C). Rats had free access to Purina Rat Chow (Purina. México) as well as tap water. The experimental protocols were approved by the Research and Ethics Committee of our University fulfilling the domestic and international standards of animal welfare including the Mexican Standards Related to Use and Management of Laboratory Animals (DOF. NOM-062-Z00-1999) as well as the National Institutes of Health (NIH publication No. 80–23, revised 1996). During the whole study, efforts to minimize animal suffering were considered and for ethical reasons, a reduced number of animals was used.

#### Chemicals

AA-5-HT was provided by Prof. Vincenzo Di Marzo and chemical was dissolved in a vehicle (VEH) solution composed of polyethylene-glycol/saline (5:95 v/v) as described previously (Murillo-Rodriguez et al., 2003, 2011a, 2013). All reagents, chemicals, and materials were purchased from Sigma–Aldrich (St Louis, MO, United States).

#### Sleep-Recording Surgeries

Animals (*n* = 25) were anesthetized with a mixture of acepromazine (0.75 mg/Kg; i.p.), xylazine (2.5 mg/Kg; i.p.), and ketamine (22 mg/Kg; i.p.) and placed in a stereotaxic frame (David Kopf Instruments, Tujunga, CA, United States) for standard sleep-recording electrodes implantation. Briefly, two stainless-steel screw electrodes were inserted 2 mm on either side of the sagittal sinus and 3 mm anterior to Bregma (frontal cortex) whereas other two screws were located 3 mm on either side of the sagittal sinus and 6 mm behind Bregma (occipital cortex). This electrode setting recorded the electroencephalogram (EEG) signal by obtaining the bipolar (differential) EEG signal recorded from two contralateral screw electrodes (frontal–occipital). In addition, the recording of the electromyogram (EMG) consisted in the signal obtained by the implantation of two wire electrodes into the dorsal neck muscles in each rat. Next, EEG/EMG wires were inserted into a six-pin plastic plug (Plastics One, Roanoke, VA, United States) and the whole electrode setting was secured onto the skull by commercial dental cement. Upon completion of the EEG/EMG surgeries, all animals were placed into individual cages with regular bedding and standard water and food containers for post-surgery recuperation period. Rats with implanted electrodes were connected to a 6-channel slip-ring commutator through a 50 cm cable (Plastics One, Roanoke, VA, United States), which allowed to the animals to free turning and moving around in the box during the whole study. The surgical procedure as well as habituation conditions (during 7 days) were carried out as reported elsewhere (Murillo-Rodríguez et al., 2011b).

#### Sleep Recordings Analysis

The EEG/EMG signals were scored in 12 s epochs which in turn allow to character the sleep-wake cycle in W, SWS, or REMS by the sleep-scoring program (ICELUS). In detail: The EEG/EMG wires were connected to the slip-ring system (Plastics One, Roanoke, VA, United States) and to the amplifier allowing to filter the EEG signal at 70 Hz (low-pass filter) and 0.3 Hz (high-pass filter; Model M15LT 15A54; Grass Instruments. Quincy, MA, United States). The EEG signals were continuously sampled at 128 Hz by using a 100-bit analog-to-digital converter board (NI PCI-6033E Multifunction I/O Board and NI-DAQ Software, SCB-100 Shielded Connector Block. National Instruments. Austin, TX, United States). With the aid of the sleep-scoring software (ICELUS), the sleep-wake cycle was characterized as follows: The presence of desynchronized EEG as well as high EMG activity belong to the definition of W whereas high-amplitude slow waves with a low EMG tone relative to awake belong to SWS criteria. At last, REMS was characterized by regular theta activity across the EEG coupled with low EMG accompanied with myoclonic features. The sleep-recordings procedures were followed as previously reported (Murillo-Rodríguez et al., 2006, 2007b, 2008b,c; Mijangos-Moreno et al., 2016).

#### Pharmacological Administrations

Right after 7 days of post-surgery and the respective habituation period, animals were placed into one of the experimental light-dark cycle block: Lights-on period (07:00–19:00 h) or lights-off period (19:00–07:00 h). According to the respective experimental light-dark block, animals were disconnected from the sleep-recording system and experimental trials were administered randomly as follows: VEH (*n* = 5), AA-5-HT (5, 10, 20 mg/Kg, i.p.; *n* = 5 each group). Irrespective of whether the injection of VEH could modify the sleep-wake cycle, an additional group named “sham” (*n* = 5) was included in the light-dark period. This group consisted in animals that received the insertion (i.p.) of the needle but no administration was given. Once experimental challenges were applied, animals were reattached to the sleep-recording system and sleep data were collected across the following 4h. In the current experiment, treatments were done randomly by using a single-blind Latin Square Experimental Design.

#### Power Spectra Analysis

Besides the effects of AA-5-HT on sleep, Fast Fourier Transformation Analysis was collected during either lights-on or lights-off cycle after respective treatments for analysis of qualitative sleep parameters such as alpha, delta and theta power spectra. Several reports have suggested that power spectra are a critical element to circumscribe discrepancies among objective and subjective measures of sleep-wake cycle (Tobler and Borbély, 1990; Franken et al., 1991; Corsi-Cabrera et al., 2001; Christensen et al., 2015; Jing et al., 2016; Imperatori et al., 2016; Svetnik et al., 2017). Thus, following standardized procedures, power spectra for alpha (during W, α = 8–12 Hz), delta (across SWS, Δ = 0.5–4.0 Hz) or theta (for REMS, Θ = 6.0–12.0 Hz) were analyzed after the experimental trials as previously reported (Murillo-Rodríguez et al., 2011a).

#### Statistical Analysis

Data from sleep studies and power spectra experiments were represented as mean ± standard error of the mean. Statistical differences were determined by one-way ANOVA followed by Scheffé’s *post hoc* test for multiple comparisons among the experimental groups. All statistical analyses were performed using the StatView (version 5.0.0, SAS Institute, United States) and statistical differences among groups were determined if *P* < 0.05.

### Experiment 2: Effects on the Extracellular Levels of Monoamines or AD after Administrations of AA-5-HT during the Lights-Off Period

#### Experimental Animals, Chemicals, and Pharmacological Administrations

As described in Experiment 1.

#### Microdialysis Surgeries

Multiple experiments have suggested the key role of nucleus accumbens (AcbC) in sleep modulation (Lazarus et al., 2012, 2013; Qiu et al., 2012; Zhang et al., 2013; Liu et al., 2016). Furthermore, previous studies from our laboratory have indicated reliable measurements of monoamines as well as AD from AcbC (Murillo-Rodríguez et al., 2006; Mijangos-Moreno et al., 2014, 2016). Thus, these evidences allowed us to collect microdialysis samples from AcbC to determine whether sleep changes provoked by AA-5-HT might be linked with effects in neurotransmitters contents. To achieve this goal, a new set of animals (*n* = 25) was anesthetized and mounted into the stereotaxic frame (David Kopf Instruments. Tujunga, CA, United States) for implantation of a microdialysis guide-cannula (IC guide; BioAnalytical Systems [BAS], West Lafayette, IN, United States). The cannula was unilaterally placed into the AcbC (coordinates: A = +1.2 and L = +2.0, H = –7.0 mm, with reference to Bregma [Paxinos and Watson, 2005]) and right after the surgery, rats were placed individually into the microdialysis bowl (Raturn Microdialysis Stand-Alone System. MD-1404, BAS. West Lafayette, IN, United States) for recovery as well as habituation for the experimental conditions across 7 days. All surgical procedures of microdialysis probes were accomplished as previously reported (Murillo-Rodríguez et al., 2007a; Mijangos-Moreno et al., 2014, 2016).

#### Microdialysis Sampling Procedures

Seven days after microdialysis surgery, the rats were removed from microdialysis bowls and the stylet from guide-cannula was withdrawn. Later, the microdialysis probe (1 mm of length; polyacrylonitrile, MWCO = 30,000 daltons; 340 μm OD; BAS. West Lafayette, IN, United States) was inserted at 07:00 h. Next, through a minitube (0.65 mm OD × 0.12 mm ID; BAS, West Lafayette, IN, United States) attached to a 2.5 mL syringe (BAS, West Lafayette, IN, United States) and using a pump (flow rate: 0.25 μL/min; BAS Bee, West Lafayette, IN, United States), artificial cerebrospinal fluid [aCSF (composition: NaCl 147 mM, KCl 3 mM, CaCl 1.2 mM, MgCl 1.0 mM, pH 7.2)] was injected. Importantly, previous experiments have found that after insertion, the microdialysis probe requires approximately 6–7 h for stabilization (Porkka-Heiskanen et al., 2000; Murillo-Rodriguez et al., 2004). However, in the current report, probes were stabilized during a total time of 24 h. Later, and once stabilization period was achieved, microdialysis samples were collected hourly across 4h from each animal after experimental trials. Next, dialysates were averaged over the four time points as a total value for each compound in every experimental condition [Sham (*n* = 5), VEH (*n* = 5), AA-5-HT (5, 10, or 20 mg/Kg, separately all doses (*n* = 5, each dose))]. It is worthy to mention that the microdialysis probes were used no more than 5 days since preceding reports have shown that subsequently insertions, gliosis significantly diminishes the membrane ability to transport fluid across the pores (Porkka-Heiskanen et al., 2000; Murillo-Rodriguez et al., 2004). Complementary, the recovery test of microdialysis probes consisted in insertion of probes into a test solution containing external known concentrations of monoamines or AD with perfusion of aCSF at working flow rate (0.25 μL/min). Microdialysates were collected under this experimental condition after triplicate using a different lot of microdialysis probes. Each sample collected from *in vitro* recovery study was analyzed and the peak area ratio was calculated against the known standards of neurotransmitters of interest. For obtaining the recovery rate, data were calculated as follows: Recovery rate (%) = (the peak area ratio of the sample from microdialysis sample)/(the peak area ratio of the sample in the test solution). The whole microdialysis procedures were carried out as previous reports (Porkka-Heiskanen et al., 2000; Blanco-Centurion et al., 2006; Mijangos-Moreno et al., 2014, 2016; Murillo-Rodríguez et al., 2016).

#### Pharmacological Challenge

Seven days after microdialysis surgery and 24 h after stabilization of microdialysis probes, experimental challenges were given to animals as follows: Due to AA-5-HT induced sleep (see Results section), animals were disconnected from microdialysis system and treatments were randomly given during the lights-off period. Right after the experimental trials were provided, rats were reattached to the microdialysis system, placed them back into the microdialysis bowls and sampling were collected across 4 h. Later, all samples were stored (-80°C) for further analysis.

#### High-Performance Liquid Chromatography (HPLC) Procedure

The collected dialysates were injected into HPLC for detection and quantification of DA, NE, EP, 5-HT, and AD. In detail: The dialysates were filtered (Millipore 0.22 μm; Merck Millipore. Darmstadt, Germany) and injected into HPLC (Modular Prominence, Shimadzu. Japan). The mobile phase for monoamines consisted in monosodium phosphate [(7 mM, pH 3.0), plus methanol (3.5%)] perfused with a pump (LC-20AT, Shimadzu, Japan) at flow rate of 80 μL/min. Separation of molecules was achieved by using a microbore column [octadecyl silica (3 μm, 100 × 1 mm), BAS, West Lafayette, IN, United States] with temperature controlled (22°C; oven CTO-20A. Shimadzu, Japan). Samples were automatically injected (SIL-20A HT Prominence HPLC, Shimadzu. Japan) into the HPLC and chromatographic data were stored on a personal computer (via computer controller CBM-20A, Shimadzu, Japan). To determine the concentrations of DA, NE, EP, 5-HT, a series of dilutions of known external standards of these neurotransmitters were prepared and injected into HPLC. To ensure the specificity of HPLC method, further procedures were carried out as suggested by the manufacturer as well as reported by others (Sartori et al., 2008; Mazzucchelli et al., 2011). Monoamines were disclosed by using an electrochemical detector (LC-4C; BAS, West Lafayette, IN, United States) as described by others (Gold et al., 2011; Grupe et al., 2013). On the other hand, AD detection required mobile phase prepared with 10 mM sodium dihydrogen phosphate (pH 4.5) and methanol (9%), infused at a flow rate of 80 μL/min using a pump (LC-20AT. Prominence HPLC, Shimadzu, Japan). Detection of AD was carried out by using an UV detector (SPD-20A Prominence. Shimadzu, Japan) set to a wavelength of 254 nm (deuterium lamp). All chromatographic data for AD detection were recorded in a personal computer, and peak heights of AD in dialysates were compared with external standards using chromatograph report software (LC Solution, Shimadzu, Japan). The procedure for detection and measurement of monoamines or AD using HPLC means was developed according to previous reports (Murillo-Rodríguez et al., 2016).

#### Histological Verification of Probe Location

After microdialysis experiments, all rats were sacrificed with a lethal dose of pentobarbital for the standard procedure for the vascular perfusion. Animals were transcardially perfused through a ventricular catheter placed on the left ventricle. Next, the right atrium was cut open and saline solution (0.9%) was perfused followed by formaldehyde (4%). The brain was removed and post-fixed overnight in formaldehyde (4%) followed by 10, 20, and 30% sucrose/PBS (0.1 M) for 24 h each concentration. At last, all brains were cut in coronal sections (20 μm) using a Portable Bench-top Cryostat (Leica CM1100. Germany) and collected in 1:5 serial order. One serial was used for probe location and it was identified by plotting using rat brain atlas (Paxinos and Watson, 2005).

#### Statistical Analysis

As described in Experiment 1.

### Experiment 3: AA-5-HT Blocks the Wake-Inducing Properties of Cannabidiol or Modafinil during the Lights-On Period

#### Experimental Animals, Chemicals, and Pharmacological Administrations

As described in Experiment 1.

#### Pharmacological Administrations

To provide further evidence regarding the sleep-inducing properties of AA-5-HT, we tested whether this compound would block the waking induced by pharmacological means. In this section of the study, administrations were given as described in Experiment 1. However, we included in this section of the study, the highest dose of AA-5-HT (20 mg/Kg; i.p.) as well as the wake-inducing drug cannabidiol (CBD, 30 mg/Kg; i.p) or the stimulant compound modafinil (MOD, 30 mg/Kg; i.p). To evaluate whether AA-5-HT would prevent the alertness induced by either CBD or MOD, the FAAH/TRPV1 modulator was administered (5, 10, or 20 mg/Kg; *n* = 5, each group) 15 min before injection of either MOD or CBD (30 mg/Kg; each separately; *n* = 5, each group).

#### Power Spectra Analysis and Statistical Analysis

The analysis of power spectra after coadministrations of either AA-5-HT + CBD or AA-5-HT + MOD and further statistical analysis were carried out as described in Experiment 1.

### Experiment 4: Effects on the Extracellular Levels of Monoamines or AD Induced by Cannabidiol or Modafinil during the Lights-On Period Are Prevented by AA-5-HT Injection

#### Experimental Animals, Chemicals, Microdialysis Surgeries, Microdialysis Sampling Procedures, HPLC Procedures, Histological Verification of Probe Location, and Statistical Analysis

As described in Experiments 2 and 3. For studies when AA-5-HT was administered before either injection of CBD or MOD, statistical differences were determined by two-way ANOVA followed by Scheffé’s *post hoc* test for multiple comparisons among the experimental groups. All statistical analyses were performed using the StatView (version 5.0.0, SAS Institute, United States) and statistical differences among groups were determined if *P* < 0.05.

#### Pharmacological Challenges

The analysis of extracellular levels of DA, NE, EP, 5-HT, and AD after coadministrations of either AA-5-HT + MOD or AA-5-HT + CBD were carried out as described in Experiments 3 and 4.

### Experiment 5: The Blockade of Sleep Rebound Caused by Either Cannabidiol or Modafinil after Total Sleep Deprivation Is Prevented by AA-5-HT

#### Experimental Animals, Chemicals, and Sleep-Recording Surgeries

As described in Experiments 1–4.

#### Total Sleep-Deprivation Procedure

Prolonged waking was carried out by maintaining rats on constant continuous alertness across 6 h during the lights-on period (from 07:00–13:00 h) by placing novel objects into the cages, generating sounds, knocking the outer walls of the cages, etc. (Mijangos-Moreno et al., 2015). During total sleep deprivation (TSD, 6 h), sleep data were constantly recorded and analyzed as reported (Murillo-Rodríguez et al., 2016).

#### Pharmacological Administrations

To test whether AA-5-HT would prevent the blocking effect on sleep rebound after prolonged waking in CBD-treated or MOD-injected rats, right after the end of TSD, rats were disconnected from the sleep-recording system and experimental trials were administered randomly as follows during the lights-on period: Sham (*n* = 5), VEH (*n* = 5), AA-5-HT (5, 10, 20 mg/Kg, i.p.; *n* = 5, each compound), CBD (30 mg/Kg, i.p.), MOD (30 mg/Kg, i.p.), AA-5-HT (20 mg/Kg, i.p.) and 15 min later CBD (30 mg/Kg, i.p.) or MOD (30 mg/Kg, i.p.). Once injections were performed, rats were reattached to the sleep-recording system, and sleep data were collected across the following 4h.

#### Power Spectra and Statistical Analysis

As described in Experiment 4.

### Experiment 6: The Blockade of the Compensatory Rebound of the Extracellular Levels of Monoamines or AD after Total Sleep Deprivation by Cannabidiol or Modafinil Is Prevented by AA-5-HT during the Lights-On Period

#### Experimental Animals, Chemicals, Microdialysis Surgeries, Microdialysis Sampling Procedures, HPLC Procedures, Histological Verification of Probe Location, Total Sleep-Deprivation, Pharmacological Challenges Procedure and Statistical Analysis

As described in Experiments 1–5.

## Results

### Experiment 1: Effects on Sleep after Injections of AA-5-HT during Either the Lights-On or Lights-Off Period

**FIGURE 1:**
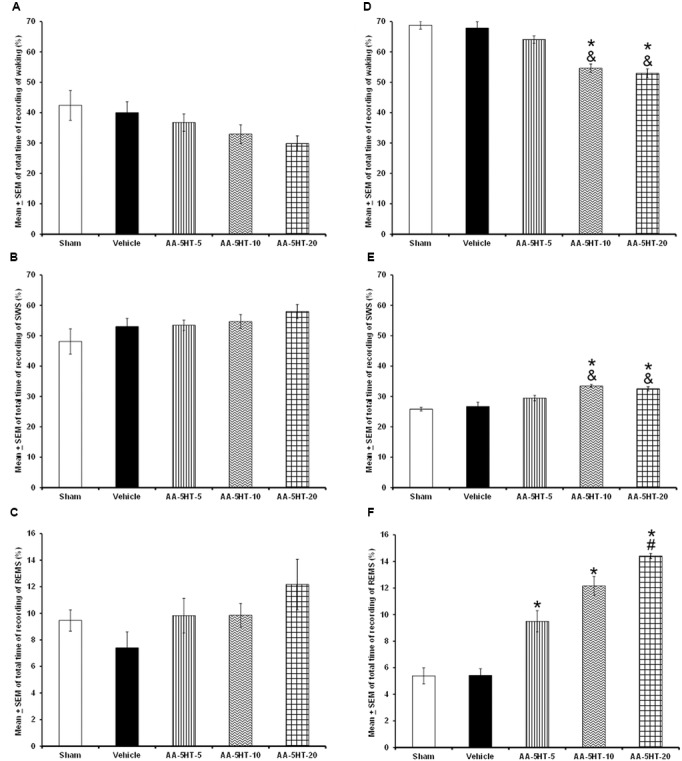
**Effects on total time (4 h of sleep recordings) of wakefulness (W), slow wave sleep (SWS), and rapid eye movement sleep (REMS) after experimental trials: Sham, vehicle or AA-5-HT (5, 10, or 20 mg/Kg; i.p.)**. No effects were observed in W, SWS, or REMS when AA-5-HT was injected during the lights-on period (**A–C**, respectively). However, when administered at the beginning of the lights-off period, AA-5-HT decreased W and increased SWS and REMS (**D–F**, respectively; Mean ± SEM ^∗^ vs. Sham/Vehicle, *P* < 0.05; & vs. AA-5-HT-5, *P* < 0.05; # vs. AA-5-HT-10, *P* < 0.05). Abbreviations: wakefulness (W), slow wave sleep (SWS) and rapid eye movement sleep (REMS), AA-5-HT-5 mg/Kg (AA-5-HT-5), AA-5-HT-10 mg/Kg (AA-5-HT-10), AA-5-HT-20 mg/Kg (AA-5-HT-20).

In the first experiment, AA-5-HT given during the lights-on period, caused no significant differences in the sleep-wake cycle between experimental groups (W: *F*_(4,20)_ = 2.062, *P* = 0.1; SWS: *F*_(4,20)_ = 1.561, *P* = 0.2; REMS: *F*_(4,20)_ = 1.614, *P* = 0.2; **Figures 1A–C**, respectively). However, AA-5-HT administered at the beginning of the lights-off period, decreased W (*F*_(4,20)_ = *7*.991, *P <* 0.0005) and enhanced SWS (*F*_(4,20)_ = 9.425*, P <* 0.05) as well as REMS (*F*_(4,20)_ = 8.324*, P <* 0.0004, **Figures 1D–F**, respectively). The Scheffé’s *post hoc* test showed statistical differences among sham/vehicle and AA-5-HT (5, 10, 20 mg/Kg) for waking, SWS and REMS during the lights-off period (*P* < 0.01). From the data obtained, we conclude that AA-5-HT caused sleep promotion if administered during the lights-on period.

**FIGURE 2:**
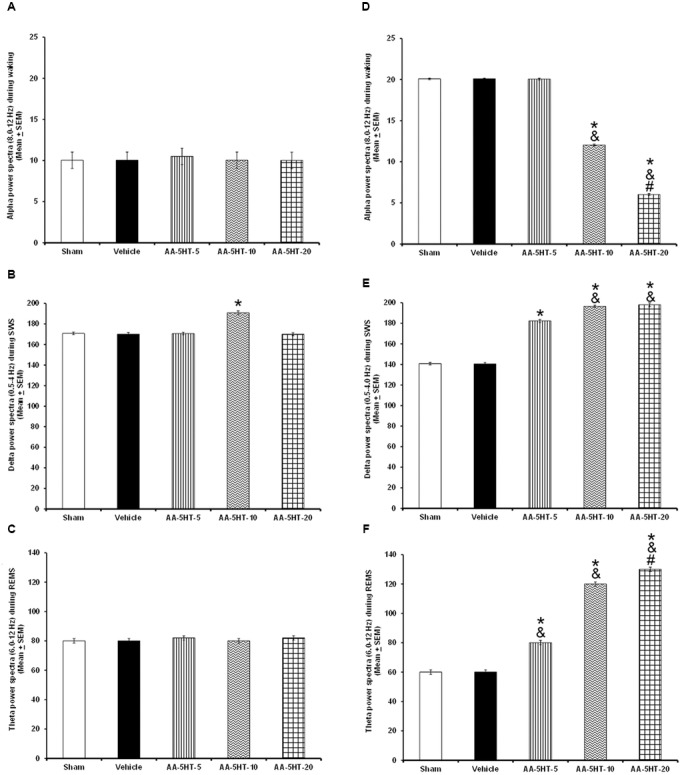
**Effects on EEG power spectra (alpha, delta or theta) after experimental trials: Sham, vehicle or AA-5-HT (5, 10, or 20 mg/Kg; i.p.)**. No effects were observed in alpha or theta power spectra when AA-5-HT was injected during the lights-on period. **(A–C)** respectively). Next, when AA-5-HT was administered at the beginning of the lights-off period, the compound decreased alpha (for W: α = 8–12 Hz), and enhanced delta (for SWS: Δ = 0.5–4.0 Hz) as well as theta (for REMS: Θ = 6.0–12.0 Hz; **(D–F)**. Mean ± SEM ^∗^ vs Sham/Vehicle, *P* < 0.05; & vs. AA-5-HT-5, *P* < 0.05; # vs. AA-5-HT-10, *P* < 0.05). Abbreviations: slow wave sleep (SWS) and rapid eye movement sleep (REMS), AA-5-HT-5 mg/Kg (AA-5-HT-5), AA-5-HT-10 mg/Kg (AA-5HT-10), AA-5-HT-20 mg/Kg (AA-5-HT-20).

Regarding the power spectra analysis (alpha collected during W, delta obtained during SWS and theta recorded during REMS), we found that systemic injections during the lights-on period of different doses of AA-5-HT (5, 10, or 20 mg/Kg, i.p.) did not modify the values for alpha (*F*_(4,20)_ = 0.964, *P* = 0.4) or theta (*F*_(4,20)_ = 1.524, *P* = 0.2) whereas enhanced delta power spectra (*F*_(4,20)_ = 3.053, *P <* 0.02; **Figures 2A–C**, respectively). When AA-5-HT was administered at the beginning of the lights-off period, a decrease in a dose-dependent fashion in alpha was found (*F*_(4,20)_ = 3.12*, P <* 0.03) while an increase in delta (*F*_(4,20)_ = 3.377*, P <* 0.02) as well as a dose-dependent enhancement in theta power spectra were observed (*F*_(4,20)_ = 3.456, *P <* 0.02; **Figures 2D–F**, respectively). The Scheffé’s *post hoc* test displayed inter-group differences among sham/vehicle and AA-5-HT (5, 10, 20 mg/Kg) for alpha, delta, and theta power spectra (*P* < 0.02). Thus, we found that AA-5-HT caused a dose-dependent effect in sleep power spectra by decreasing alpha and enhancing theta power spectra. We conclude that diminution observed in waking likely corresponds to the decrease found in alpha power spectra. Conversely, the enhancement in SWS and REMS caused by AA-5-HT is accompanied by an increase in delta and theta power spectra, respectively.

### Experiment 2: Effects on the Extracellular Levels of Monoamines or AD after Administrations of AA-5-HT during the Lights-Off Period

**FIGURE 3:**
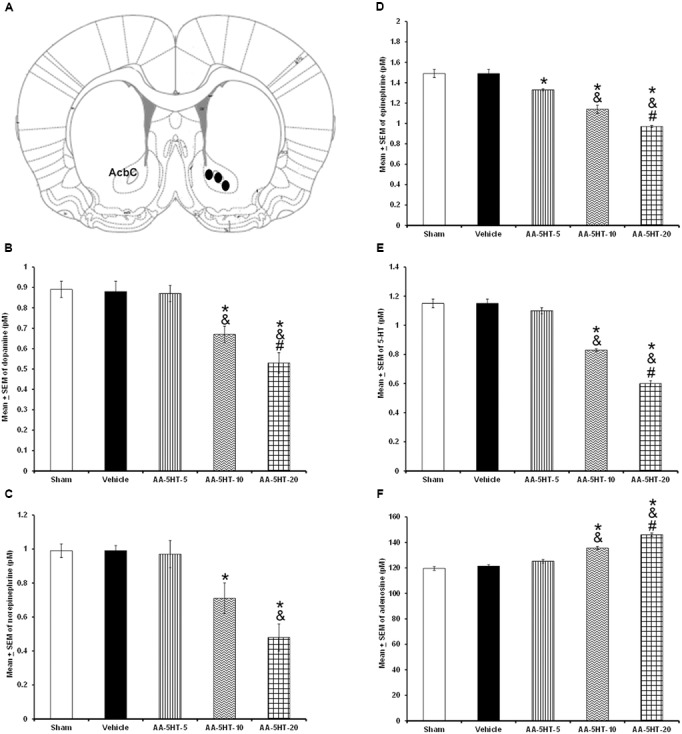
**Effects of administration of AA-5-HT (5, 10, or 20 mg/Kg; i.p.) during the lights-off period on the extracellular levels of dopamine (DA), norepinephrine (NE), epinephrine (EP), serotonin (5-HT), and adenosine (AD)**. Schematic representation of position of the microdialysis probe into AcbC (**A**- Black dots represent the tip of microdialysis probe). AA-5-HT decreased the contents of DA, NE, EP, and 5-HT whereas AD levels were enhanced (**B–F**, respectively. Mean ± SEM ^∗^ vs. Sham/Vehicle, *P* < 0.05; & vs. AA-5-HT-5, *P* < 0.05; # vs. AA-5-HT-10, *P* < 0.05). Abbreviations: AA-5-HT-5 mg/Kg (AA-5-HT-5), AA-5-HT-10 mg/Kg (AA-5-HT-10), AA-5-HT-20 mg/Kg (AA-5-HT-20). Drawing taken from Paxinos and Watson Rat Brain Atlas (2005).

In the microdialysis experiments, position of probe was confirmed as shown in **Figure 3A**. Since we observed that AA-5-HT increased sleep during the active period of the rats, in the microdialysis experiments, compound was given at the beginning of the lights-off period. After injection of AA-5-HT, we found a decrease in contents of DA (*F*_(4,20)_ = 7775.000, *P <* 0.0001), NE (*F*_(4,20)_ = 1066.582, *P <* 0.0001), EP (*F*_(4,20)_ = 873.101; *P <* 0.0001) and 5-HT (*F*_(4,20)_ = 1288.113, *P <* 0.0001) whereas AA-5-HT enhanced AD levels (*F*_(4,20)_ = 773.200, *P <* 0.0001; **Figures 3B–F**, respectively). A dose-dependent effect was observed in DA, NE, EP, 5-HT, and AD contents in AA-5-HT-treated rats. The Scheffé’s *post hoc* test showed inter-group differences between sham/vehicle and AA-5-HT (5, 10, 20 mg/Kg) for DA, NE, EP, 5-HT, and AD (*P* < 0.0001). We conclude that injection of AA-5-HT during the lights-off period reduced in a dose-dependent fashion the contents of monoamines whereas caused an opposite effect in AD levels.

### Experiment 3: AA-5-HT Blocks the Wake-Inducing Properties of Cannabidiol or Modafinil during the Lights-On Period

**FIGURE 4:**
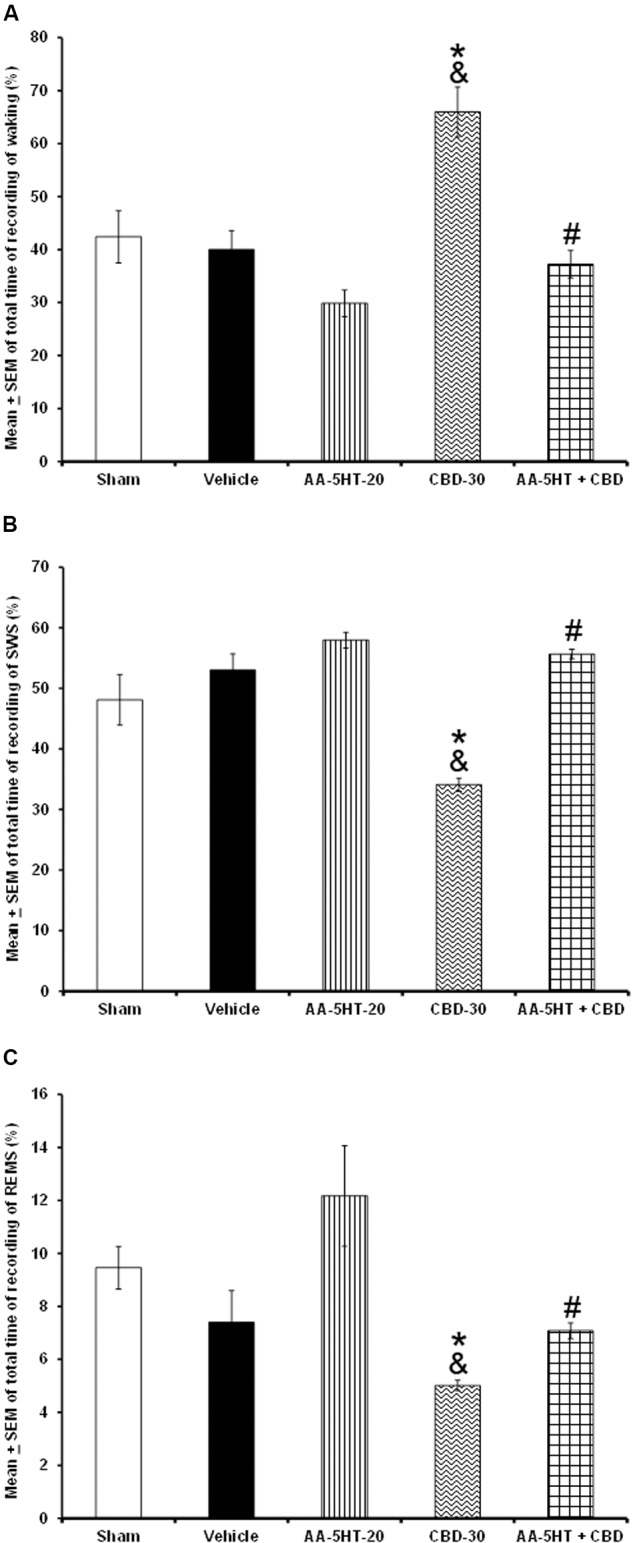
**Effects on total time of W, SWS, and REMS after experimental trials administered at the beginning of the lights-on period: Sham, vehicle, AA-5-HT (20 mg/Kg; i.p.), cannabidiol (CBD, 30 mg/Kg; i.p.) or AA-5-HT (20 mg/Kg; i.p.) and 15 min CBD (30 mg/Kg; i.p.)**. AA-5-HT caused no statistical difference in waking, SWS and REMS. However, CBD (30 mg/Kg; i.p.) enhanced alertness and decreased SWS as well as REMS. Interestingly, AA-5-HT administered 15min before the injection of CBD blocked the increase in W as well as the enhancement in SWS and partially REMS caused by CBD (**A–C**, respectively; Mean ± SEM ^∗^ vs. Sham/Vehicle, *P* < 0.05; & vs. AA-5-HT-20, *P* < 0.05; # vs. CBD-30, *P* < 0.05). Abbreviations: Cannabidiol 30 mg/Kg, i.p. (CBD-30), AA-5-HT-20 mg/Kg (AA-5-HT-20).

Next, we tested whether AA-5-HT would block the wake-inducing effects of CBD during the lights-on period. Due to ethical reasons, animals from sleep-wake study (sham, vehicle and AA-5-HT groups, **Figure 1**) were used for comparisons in this section of the experiment. As described previously, AA-5-HT caused no statistical differences in waking, SWS and REMS if injected during the lights-on period. However, administration of CBD (30 mg/Kg, i.p.) enhanced alertness and decreased SWS as well as REMS. This effects confirmed previous reports (Murillo-Rodríguez et al., 2006, 2008b). Interestingly, AA-5-HT administered 15min before the injection of CBD was able to block the increase in W (*F*_(4,20)_ = 14.502, *P <* 0.0001) as well as the enhancement in SWS (*F*_(4,20)_ = 8.372, *P <* 0.0004) and REMS (*F*_(4,20)_ = 3.675, *P <* 0.02; **Figures 4A–C**, respectively). The Scheffé’s *post hoc* test showed inter-group differences for among sham/vehicle and AA-5-HT as well as CBD and AA-5-HT + CBD for waking, SWS and REMS (*P* < 0.01). We conclude that AA-5-HT blocked the wake-inducing effects of CBD.

**FIGURE 5:**
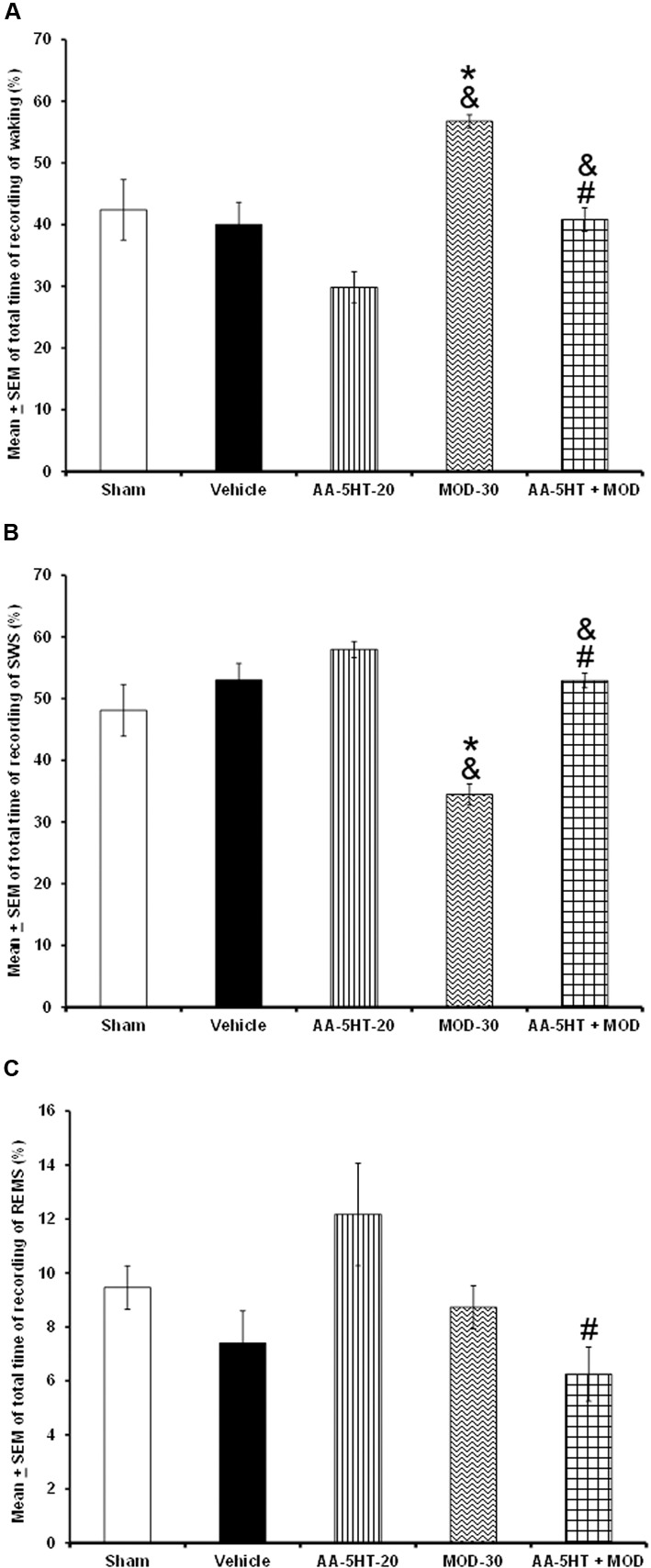
**Effects on total time of W, SWS, and REMS after experimental trials: Sham, vehicle, AA-5-HT (20 mg/Kg; i.p.), modafinil (MOD, 30 mg/Kg; i.p.) or AA-5-HT (20 mg/Kg; i.p.) and 15 min MOD (30 mg/Kg; i.p.)**. AA-5-HT caused no statistical difference in waking, SWS and REMS if injected at the beginning of the lights-on period. However, the administration of MOD (30 mg/Kg; i.p.) increased W and decreased SWS. No statistical changes were observed in REMS in MOD-treated animals. Interestingly, administration of AA-5-HT 15 min before the injection of MOD prevented the enhancement in waking as well as the diminution in SWS. Despite that MOD induced no effects in REMS, combination of AA-5-HT + MOD decreased REMS (**A–C**, respectively) induced by the stimulant (Mean ± SEM ^∗^ vs. Sham/Vehicle, *P* < 0.05; & vs. AA-5-HT-20, *P* < 0.05; # vs. MOD-30, *P* < 0.05). Abbreviations: Modafinil 30 mg/Kg, i.p. (MOD-30), AA-5-HT-20 mg/Kg (AA-5-HT-20).

Our next aim was to determine whether AA-5-HT might prevent the alertness caused by MOD if injected at the beginning of the lights-on period. Due to ethical reasons, rats from sleep-wake study (sham, vehicle, and AA-5-HT groups, **Figure 1**) were used for comparisons in this experiment. As described previously, AA-5-HT caused no statistical differences in alertness, SWS and REMS if injected at the beginning of the lights-on period. Nevertheless, the administration of MOD (30 mg/Kg, i.p.) increased W and decreased SWS. No statistical differences were found in REMS. The wake-inducing properties effects of MOD in our experiment confirmed previous observations (Scammell et al., 2000; Murillo-Rodríguez et al., 2007a, 2008c). Importantly, administration of AA-5-HT 15 min before the injection of MOD prevented the enhancement in waking (*F*_(4,20)_ = 9.941, *P <* 0.0001) as well as the diminution in SWS (*F*_(4,20)_ = 9.409, *P* < 0.0002; **Figures 5A–C**, respectively). The Scheffé’s *post hoc* test showed inter-group differences for among sham/vehicle and AA-5-HT as well as MOD and AA-5-HT + MOD for waking, SWS and REMS (*P* < 0.0001). Our data suggest that AA-5-HT blocked the increase observed in waking as well as the decrease in SWS caused by MOD.

**FIGURE 6:**
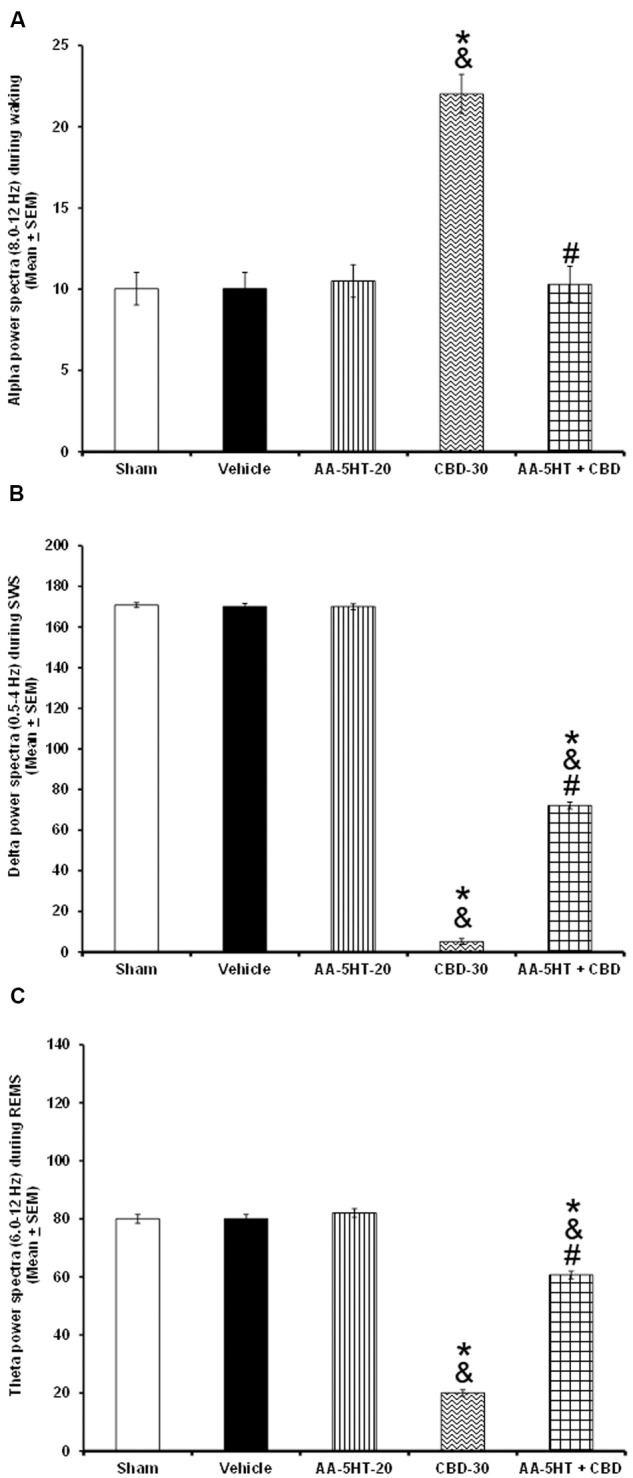
**Effects on EEG power spectra (alpha [for W: α = 8–12 Hz], delta [for SWS: Δ = 0.5–4.0 Hz] or theta [for REMS: Θ = 6.0–12.0 Hz]) after experimental trials: Sham, vehicle, AA-5-HT (20 mg/Kg; i.p.), cannabidiol (CBD; 30 mg/Kg; i.p.) or AA-5-HT (20 mg/Kg; i.p.) and 15 min CBD (30 mg/Kg; i.p.) at the beginning of the lights-on period**. Administration of AA-5-HT caused no statistical changes in power spectra. However, CBD (30 mg/Kg; i.p.) increased alpha, and decreased delta as well as theta power. Moreover, administration of AA-5HT before injection of CBD prevented the changes caused by the cannabinoid in alpha and partially delta and theta power spectra spectra (**A–C**, respectively; Mean ± SEM ^∗^ vs. Sham/Vehicle, *P* < 0.05; & vs. AA-5-HT-20, *P* < 0.05; # vs. CBD-30, *P* < 0.05). Abbreviations: Cannabidiol 30 mg/Kg, i.p. (CBD-30), AA-5-HT-20 mg/Kg (AA-5-HT-20).

In the following experiment, we evaluated if AA-5-HT would block the effects of CBD in power spectra (alpha collected during W, delta obtained during SWS and theta recorded during REMS), during the lights-on period. Due to ethical reasons, animals from power spectra experiment (sham, vehicle and AA-5-HT groups, **Figure 2**) were used for comparisons in this experiment. As showed previously, administration of AA-5-HT caused no statistical changes in power spectra if injected during the lights-on period. Notwithstanding, CBD (30 mg/Kg, i.p.) increased alpha, and decreased delta as well as theta power spectra. Moreover, administration of AA-5-HT before injection of CBD prevented the enhancement caused by the cannabinoid in alpha (*F*_(4,20)_ = 5.463, *P <* 0.002), partially prevented the diminution in delta (*F*_(4,20)_ = *5.995*, *P <* 0.002) and theta power spectra (*F*_(4,20)_ = 5.112, *P <* 0.002; **Figures 6A–C**, respectively). Further analysis of intragroup effects (Schefféś *post hoc* test) showed differences among sham/vehicle and AA-5-HT as well as CBD and AA-5-HT + CBD for alpha, delta and theta power spectra (*P* < 0.001). We conclude that AA-5-HT was able to block the increase in alpha power spectra whereas a limited preventing effect was observed in delta and theta power spectra in CBD-treated rats.

**FIGURE 7:**
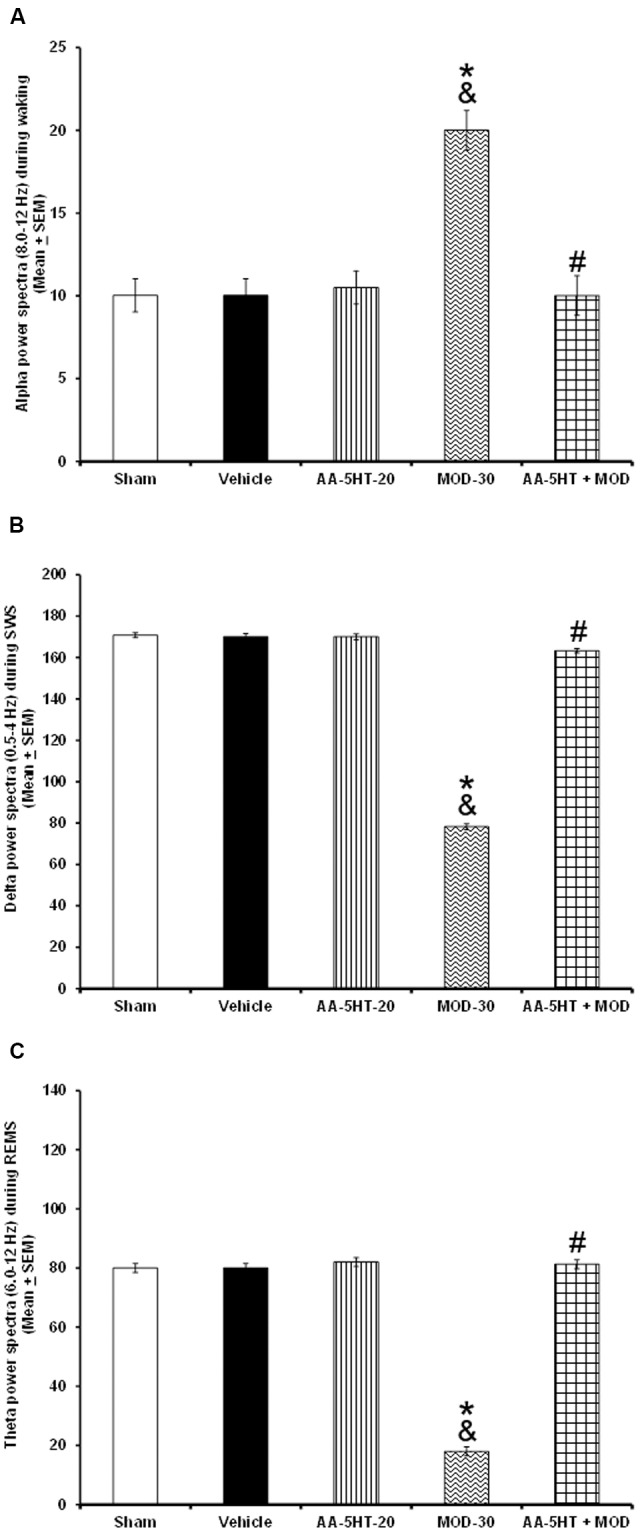
**Effects on EEG power spectra (alpha [for W: α = 8–12 Hz], delta [for SWS: Δ = 0.5-4.0 Hz], or theta [for REMS: Θ = 6.0–12.0 Hz]) after experimental trials: Sham, vehicle, AA-5-HT (20 mg/Kg; i.p.), modafinil (MOD; 30 mg/Kg; i.p.) or AA-5-HT (20 mg/Kg; i.p.) and 15 min MOD (30 mg/Kg; i.p.) at the beginning of the lights-on period**. AA-5-HT caused no statistical effects in power spectra. However, injection of MOD (30 mg/Kg; i.p.) increased alpha and decrease delta and theta power spectra. Moreover, injection of AA-5-HT before administration of MOD was able to block the enhancement in in alpha, delta and theta power spectra (**A–C**, respectively. Mean ± SEM ^∗^ vs. Sham/Vehicle, *P* < 0.05; & vs. AA-5-HT-20, *P* < 0.05; # vs. MOD-30, *P* < 0.05). Abbreviations: Modafinil 30 mg/Kg, i.p. (MOD-30), AA-5-HT-20 mg/Kg (AA-5-HT-20).

Whether AA-5-HT would be able to block the effects of MOD in power spectra during the lights-on period was evaluated in the next experiment. By reasons of ethical issues, rats from power spectra experiment (sham, vehicle and AA-5-HT groups, **Figure 2**) were used for comparisons in this section of the study. We found that AA-5-HT caused no statistical effects in power spectra. However, injection of MOD (30 mg/Kg, i.p.) during the lights-on period increased alpha and decrease delta and theta power spectra. Moreover, administration of AA-5-HT before injection of MOD blocked the enhancement in alpha (*F*_(4,20)_ = 6.872, *P <* 0.01), and prevented the decrease in delta (*F*_(4,20)_ = 4.715, *P <* 0.01) and theta power spectra (*F*_(4,20)_ = 5.501, *P <* 0.01; **Figures 7A–C**, respectively). Statistical intragroup analysis (Scheffé’s *post hoc* test) showed differences among sham/vehicle and AA-5-HT as well as MOD and AA-5-HT + MOD for alpha, delta, and theta power spectra (*P* < 0.01). In conclusion, we observed that AA-5-HT prevented the enhancement in alpha as well as the diminution in delta and theta power spectra caused by MOD injection.

### Experiment 4: Effects on the Extracellular Levels of Monoamines or AD Induced by Cannabidiol or Modafinil during the Lights-On Period Are Prevented by AA-5-HT Injection

**FIGURE 8:**
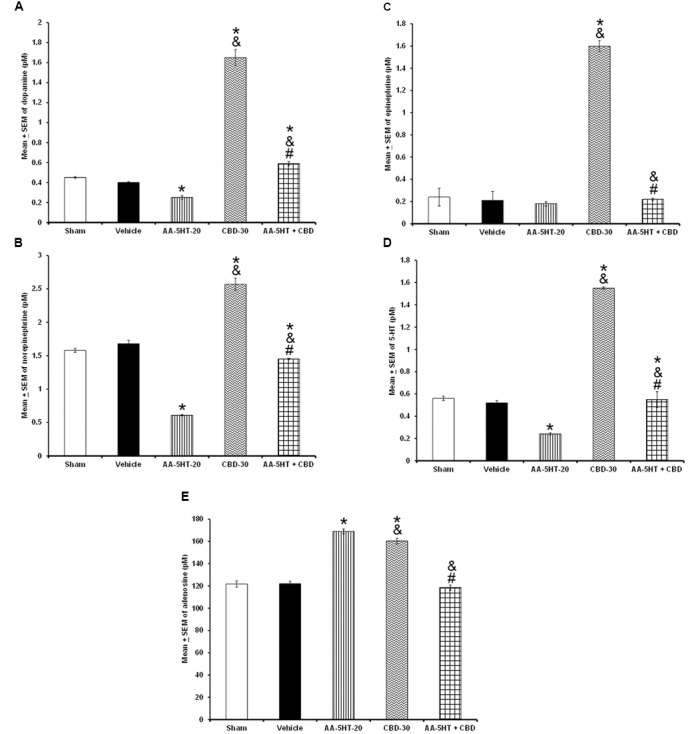
**Effects on the extracellular levels of DA, NE, EP, 5-HT, and AD after the administration of AA-5-HT (20 mg/Kg; i.p.), cannabidiol (CBD; 30 mg/Kg; i.p.) or AA-5-HT (20 mg/Kg; i.p.) and 15 min CBD (30 mg/Kg; i.p.) at the beginning of the lights-on period**. Administration of AA-5-HT decreased extracellular contents of DA, NE, 5-HT and increased AD levels as well. No statistical differences were observed in NE contents. Moreover, injection of CBD (30 mg/Kg; i.p.) increased all neurotransmitters levels. Next, the administration of AA-5-HT before injection of CBD was able to prevent the enhancement in DA, NE, EP, 5-HT as well as AD levels (**A–E**, respectively. Mean ± SE. ^∗^ vs. Sham/Vehicle, *P* < 0.05; & vs. AA-5-HT-20, *P* < 0.05; # vs. CBD-30, *P* < 0.05). Abbreviations: Cannabidiol 30 mg/Kg, i.p. (CBD-30), AA-5-HT-20 mg/Kg (AA-5-HT-20).

In the next set of experiments, we described the blocking effects of AA-5-HT on the extracellular levels of DA, NE, EP, 5-HT, and AD in response to CBD during the lights-on period. Administration of AA-5-HT decreased extracellular contents of DA, NE, 5-HT, and increased AD levels as well. No statistical differences were found in EP contents. Moreover, injection of CBD (30 mg/Kg, i.p.) increased all neurotransmitters studied. Next, the administration of AA-5-HT before injection of CBD partially prevented the enhancement in DA as shown by two-way ANOVA (*F*_(4,20)_ = 1964.342, *P* < 0.0001), and blocked the increase in NE (*F*_(4,20)_ = 451.868, *P* < 0.0001), EP (*F*_(4,20)_ = 107.976, *P* < 0.0001), 5-HT (*F*_(4,20)_ = 790.761, *P* < 0.0001) as well as the enhancement in AD levels (*F*_(4,20)_ = 843.897, *P* < 0.0001; **Figures 8A–E**, respectively). Further Scheffé’s *post hoc* test showed inter-group differences among sham/vehicle and AA-5-HT as well as CBD and AA-5-HT + CBD in levels of DA, NE, EP, 5-HT, AD (*P* < 0.0001). We conclude that injection of AA-5-HT before administration of CBD was able to block the enhancement in the levels of neurotransmitters in response to CBD administration.

**FIGURE 9:**
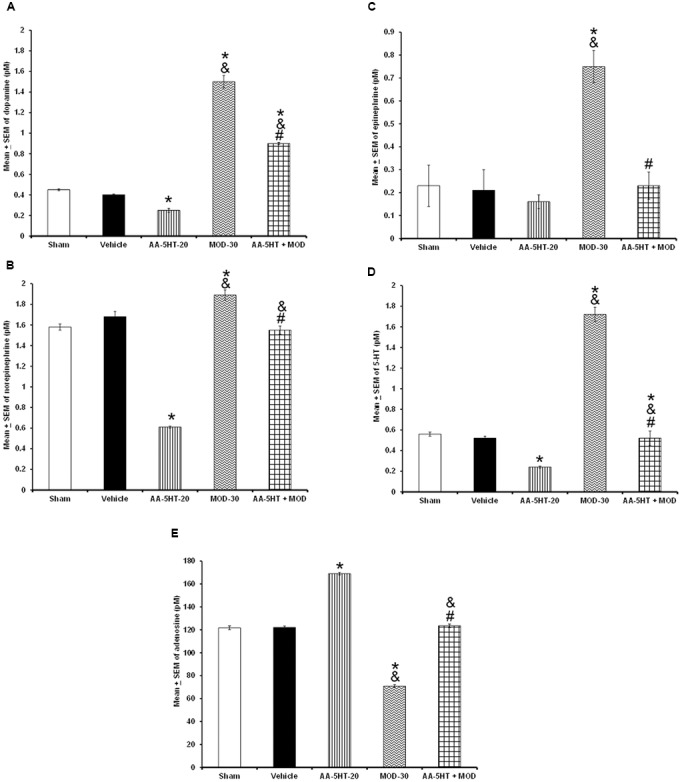
**Effects on the extracellular levels of DA, NE, EP, 5-HT, and AD after the administration of AA-5-HT (20 mg/Kg; i.p.), modafinil (MOD; 30 mg/Kg; i.p.) or AA-5-HT (20 mg/Kg; i.p.), and 15 min MOD (30 mg/Kg; i.p.) at the beginning of the lights-on period**. AA-5-HT decreased the extracellular contents of catecholamines whereas increased levels of AD. On the other hand, MOD induced opposite effects. Importantly, administration of AA-5-HT before the injection of MOD partially was able to prevent the increase in levels of DA, NE, EP, 5-HT, and blocked the decrease of AD contents (**A–E**, respectively. Mean ± SEM ^∗^ vs. Sham/Vehicle, *P* < 0.05; & vs. AA-5-HT-20, *P* < 0.05; # vs. MOD-30, *P* < 0.05). Abbreviations: Modafinil 30 mg/Kg, i.p. (MOD-30), AA-5-HT-20 mg/Kg (AA-5-HT-20).

To further characterize whether AA-5-HT administered before MOD would prevent the effects of this stimulant on the extracellular levels of DA, NE, EP, 5-HT, and AD during the lights-on period, we developed the corresponding experiment. Considering ethical reasons, animals from microdialysis study (sham, vehicle and AA-5-HT groups, **Figure 8**) were used for comparisons in this experiment. It was found that AA-5-HT decreased the extracellular contents of DA, NE, 5-HT whereas increased levels of AD. No statistical differences were found in EP extracellular contents. On the other hand, MOD induced opposite effects since enhanced extracellular levels of DA, NE, EP, 5-HT while decreased contents of AD. Importantly, administration of AA-5-HT before the injection of MOD partially prevented the increase in levels of DA as analyzed by two-way ANOVA (*F*_(4,20)_ = 1798.342, *P <* 0.0001), NE (*F*_(4,20)_ = 237.909, *P <* 0.0001), EP (*F*_(4,20)_ = 208.533, *P* < 0.0001), 5-HT (*F*_(4,20)_ = 1198.470, *P <* 0.0001) and blocked the decrease in levels of AD (*F*_(4,20)_ = 1614.168, *P <* 0.0001; **Figures 9A–E**, respectively). Further Scheffé’s *post hoc* test showed inter-group differences between sham/vehicle and AA-5-HT as well as MOD and AA-5-HT + MOD for DA, NE, EP, 5-HT, and AD (*P* < 0.0001). Based in our findings, it can be concluded that AA-5-HT prevented the effects in neurotransmitters caused by CBD injection during the lights-on period.

### Experiment 5: The Blockade of Sleep Rebound Caused by Either Cannabidiol or Modafinil after Total Sleep Deprivation Is Prevented by AA-5-HT

**FIGURE 10:**
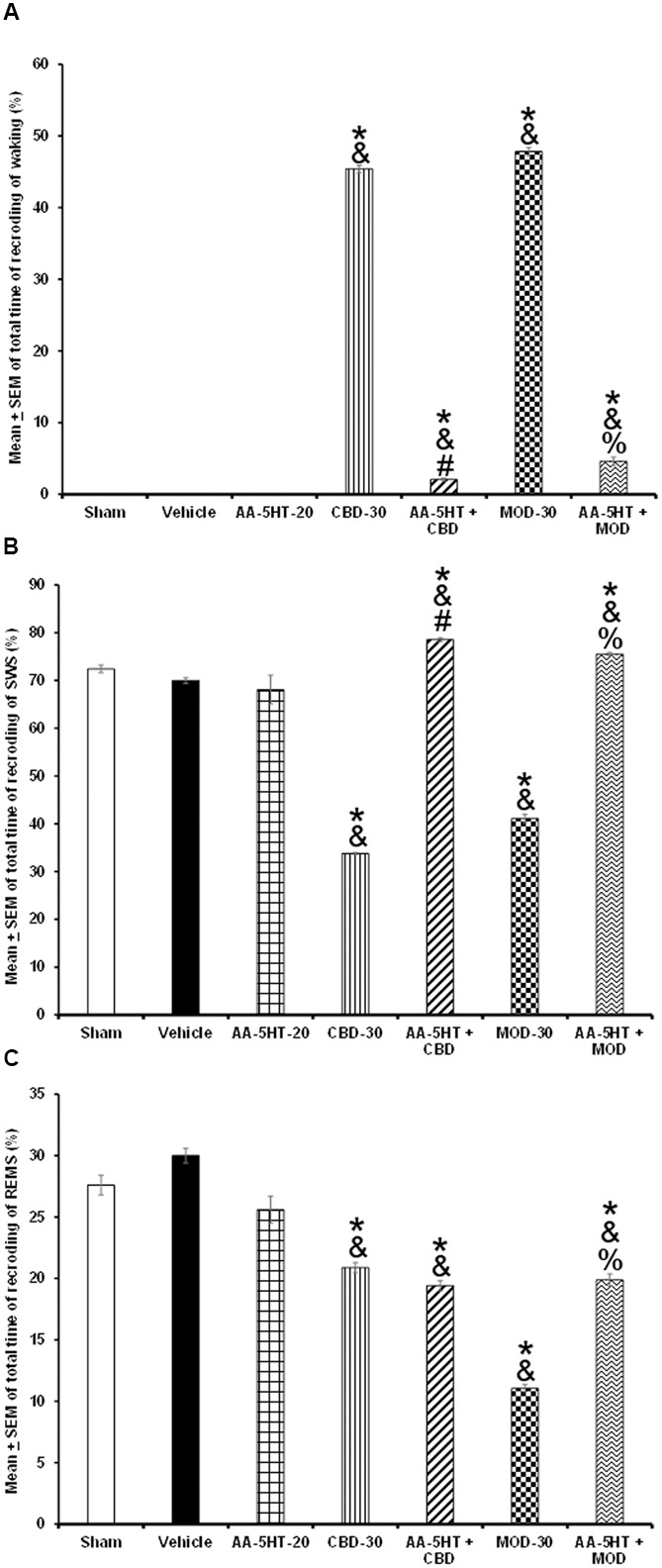
**Effects of experimental trials: Sham, vehicle, AA-5-HT (20 mg/Kg; i.p.), cannabidiol (CBD; 30 mg/Kg; i.p.), AA-5-HT (20 mg/Kg; i.p.) and 15 min later CBD (30 mg/Kg; i.p.), modafinil (MOD; 30 mg/Kg; i.p.) or AA-5-HT (20 mg/Kg; i.p.) and 15 min later MOD (30mg/Kg; i.p.) on W, SWS, or REMS during the sleep rebound period in sleep-deprived rats**. AA-5-HT caused no statistical changes in W, SWS or REMS after total sleep deprivation. Moreover, during the sleep rebound period, CBD or MOD increased waking and decreased SWS and REMS. Interestingly, during the sleep rebound period, the administration of AA-5-HT before injection of CBD prevented the increase in W as well as the decrease in SWS and partially REMS in CBD-treated rats. Similar results in sleep were obtained when AA-5-HT was administered before MOD after total sleep deprivation (**A–C**, respectively. Mean ± SEM ^∗^ vs. Sham/Vehicle, *P* < 0.05; & vs. AA-5HT-20, *P* < 0.05; # vs. CBD-30, *P* < 0.05; % vs. MOD-30, *P* < 0.05). Abbreviations: Cannabidiol 30 mg/Kg, i.p. (CBD-30), modafinil 30 mg/Kg, i.p. (MOD-30), AA-5-HT-20 mg/Kg (AA-5-HT-20).

As shown previously in the current report, AA-5-HT promoted sleep during the active period of rats and decreased wake-related neurotransmitters. Moreover, this drug blocked the wake-promoting effects of CBD or MOD. However, to characterize the properties of AA-5-HT in sleep homeostasis, we explored the possibility that the blockade by CBD or MOD of the compensatory sleep rebound after TSD would be prevented by injection of AA-5-HT during the lights-on period. We found that AA-5-HT caused no statistical changes in W, SWS or REMS if given after TSD. Moreover, during the sleep rebound period, CBD or MOD increased waking and decreased SWS and REMS. Interestingly, during the sleep rebound period, the administration of AA-5-HT before injection of CBD partially prevented the increase in W as shown by two-way ANOVA (*F*_(6,28)_ = 6063.477; *P <* 0.0001) as well as the decrease in SWS (*F*_(6,28)_ = 3649.311; *P <* 0.0001) and REMS (*F*_(6,28)_ = 1050.707; *P <* 0.0001; **Figures 10A–C**, respectively). Similar results were obtained when AA-5-HT was administered before MOD during sleep rebound period. Further Scheffé’s *post hoc* test showed inter-group differences among sham/vehicle and AA-5-HT as well as CBD, MOD, AA-5-HT + CB as well as AA-5-HT + MOD for alertness, SWS and REMS (*P* < 0.0001). We conclude that AA-5-HT was partially able to prevent the enhancement in W as well as the decrease in SWS or REMS caused by either CDB or MOD during sleep rebound period after TSD.

**FIGURE 11:**
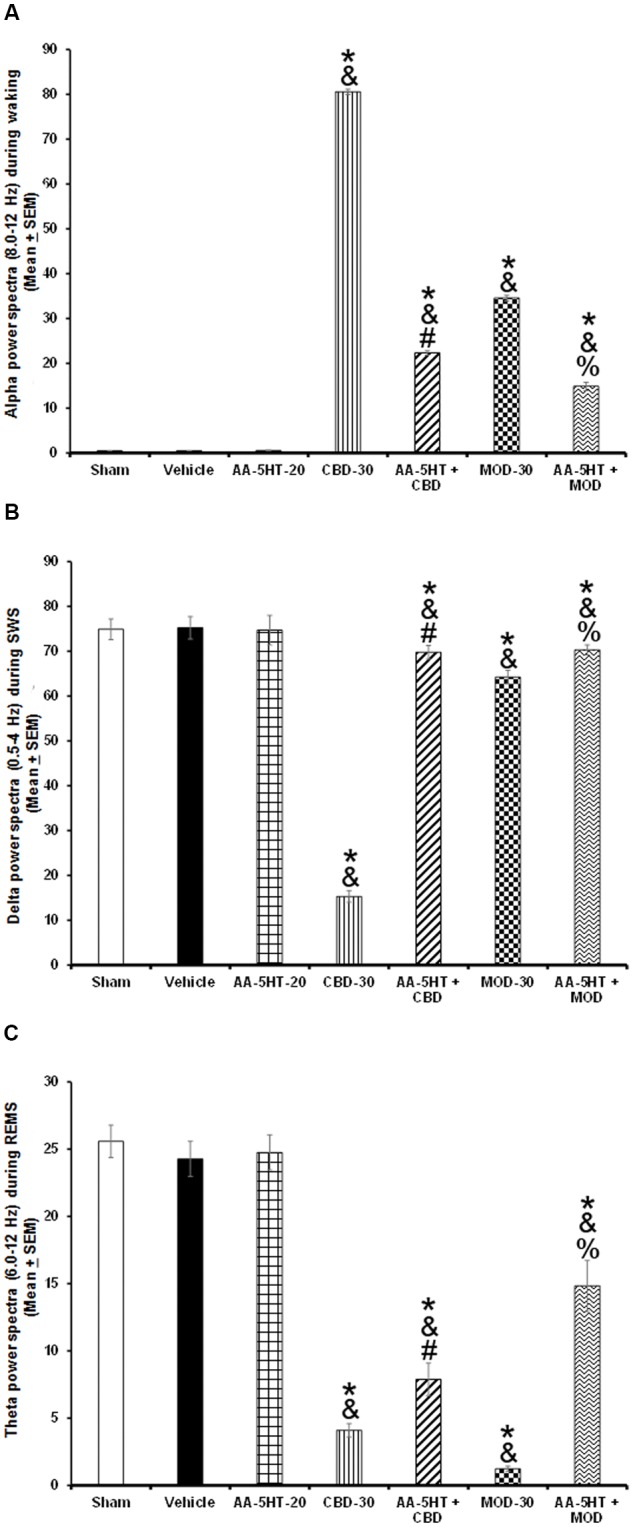
**Effects of experimental trials: Sham, vehicle, AA-5-HT (20 mg/Kg; i.p.), cannabidiol (CBD; 30 mg/Kg; i.p.), AA-5-HT (20 mg/Kg; i.p.) and 15 min later CBD (30 mg/Kg; i.p.), modafinil (MOD; 30 mg/Kg; i.p.) or AA-5-HT (20 mg/Kg; i.p.) and 15 min later MOD (30 mg/Kg; i.p.) during the sleep rebound period in sleep-deprived rats on power spectra alpha (for W: α = 8–12 Hz), delta (for SWS: Δ = 0.5–4.0 Hz) or theta power spectra (for REMS: Θ = 6.0–12.0 Hz)**. AA-5-HT caused no effects in power spectra. However, CBD or MOD partially increased alpha and decreased delta and theta power spectra. Importantly, the administration of AA-5-HT before injection of either CBD of MOD prevented the increase in alpha as well as blocked the diminution in delta and theta (**A–C**, respectively. Mean ± SEM ^∗^ vs. Sham/Vehicle, *P* < 0.05; & vs. AA-5-HT-20, *P* < 0.05; # vs. CBD-30, *P* < 0.05; % vs. MOD-30, *P* < 0.05). Abbreviations: Cannabidiol 30 mg/Kg, i.p. (CBD-30), modafinil 30 mg/Kg, i.p. (MOD-30), AA-5-HT-20 mg/Kg (AA-5-HT-20).

Next, we evaluated the changes of power spectra after TSD in animals that received AA-5-HT, CBD, MOD or administration of AA-5-HT before either CBD or MOD. We found that AA-5-HT caused no effects in power spectra. However, CBD or MOD increased alpha and decreased delta and theta power spectra. The most prominent effect was observed in CBD-treated animals. Importantly, the administration of AA-5-HT before injection of either CBD or MOD partially diminished the increase in alpha as tested by two-way ANOVA (*F*_(6,28)_ = 4.474, *P <* 0.004), blocked the diminution in delta (*F*_(6,28)_ = 4.079, *P <* 0.004) and prevented the decrease in theta power spectra (*F*_(6,28)_ = 5.109, *P <* 0.004; **Figures 11A–C**, respectively) during sleep rebound period. Additionally, inter-group differences were found by Scheffé’s *post hoc* test for alpha, delta, and theta power spectra (*P* < 0.0001). We conclude that AA-5-HT was partially able to prevent the effects on power spectra after either CBD or MOD injection after TSD.

### Experiment 6: The Blockade of the Compensatory Rebound of the Extracellular Levels of Monoamines or AD after Total Sleep Deprivation by Cannabidiol or Modafinil Is Prevented by AA-5-HT during the Lights-On Period

**FIGURE 12:**
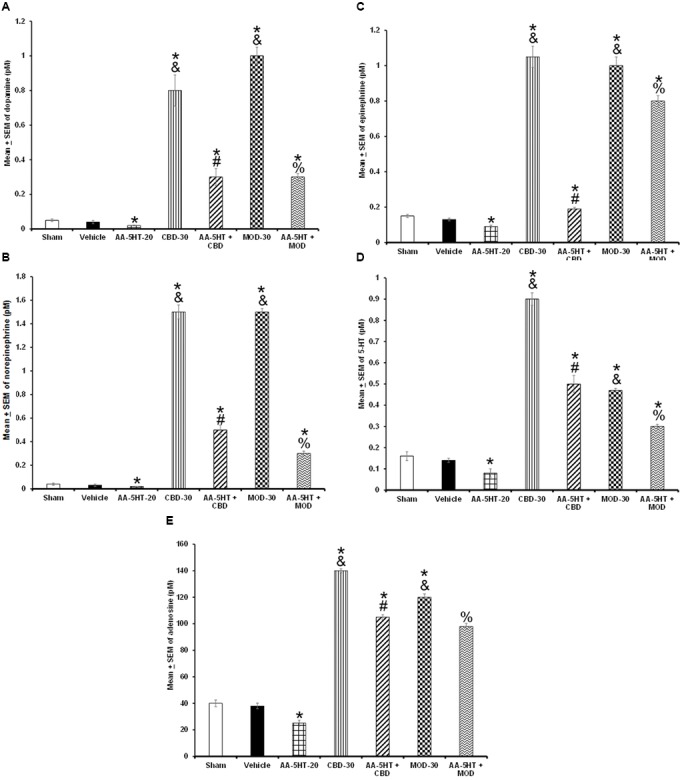
**Effects of experimental trials: Sham, vehicle, AA-5-HT (20 mg/Kg; i.p.), cannabidiol (CBD; 30 mg/Kg; i.p.), AA-5-HT (20 mg/Kg; i.p.) and 15 min later CBD (30 mg/Kg; i.p.), modafinil (MOD; 30 mg/Kg; i.p.) or AA-5-HT (20 mg/Kg; i.p.) and 15 min later MOD (30 mg/Kg; i.p.) during the sleep rebound period in sleep-deprived rats on extracellular levels of DA, NE, EP, 5-HT, and AD**. AA-5-HT decreased extracellular levels of all neurotransmitters studied. Moreover, injection of either CBD or MOD increased the contents of DA, NE, EP, 5-HT and AD (**A–E**, respectively) during the sleep rebound period. Importantly, combination of AA-5-HT + CBD partially prevented the CBD-induced changes in neurotransmitters. Similar results were observed in AA-5-HT + MOD-treated rats compared to MOD-injected animals (Mean ± SEM ^∗^ vs. Sham/Vehicle, *P* < 0.05; & vs. AA-5-HT-20, *P* < 0.05; # vs. CBD-30, *P* < 0.05; % vs. MOD-30, *P* < 0.05). Abbreviations: Cannabidiol 30 mg/Kg, i.p. (CBD-30), modafinil 30 mg/Kg, i.p. (MOD-30), AA-5-HT-20 mg/Kg (AA-5-HT-20).

In the last experiment, we investigated whether the blockade of the compensatory balance of the extracellular levels of DA, NE, EP, 5-HT, or AD after TSD by either CBD or MOD would be prevented by AA-5-HT. We found that AA-5-HT decreased after TSD the extracellular levels of neurotransmitters studied. Moreover, injection of either CBD or MOD increased the contents of DA as determined by two-way ANOVA (*F*_(6,28)_ = 1952.414, *P <* 0.0001), NE (*F*_(6,28)_ = 436.222, *P <* 0.0001), EP (*F*_(6,28)_ = 212.732, *P <* 0.0001), 5-HT (*F*_(6,28)_ = 1384.522; *P <* 0.0001) and AD (*F*_(6,28)_ = *1743.304*, *P <* 0.0001; **Figures 12A–E**, respectively) during the sleep rebound period. The most prominent effect was observed in CBD-injected rats. Moreover, inter-group differences were found by Scheffé’s *post hoc* between sham/vehicle and AA-5-HT, CBD, MOD, AA-5-HT + CB as well as AA-5-HT + MOD for DA, NE, EP, 5-HT, and AD (*P* < 0.0001). Combination of AA-5-HT + CBD partially blocked the enhancement in neurotransmitters levels in CBD-treated animals. Similar findings were observed in MOD-treated rats. We conclude that AA-5-HT did not fully block the enhancement found in neurotransmitters in animals that received either CBD or MOD during sleep rebound period after TSD.

## Discussion

The endocannabinoid system exerts multiple and complex modulatory physiological functions (Kendall and Yudowski, 2016; Ligresti et al., 2016; Argueta and DiPatrizio, 2017; Bennett et al., 2017; Dos Anjos-Garcia et al., 2017; Sun et al., 2017). For instance, cumulative evidence has suggested that the elements of the endocannabinoid system, including FAAH, control the sleep-wake cycle (Santucci et al., 1996; Murillo-Rodríguez et al., 1998, 2001, 2003, 2008a, 2011a, 2013, 2016; Herrera-Solis et al., 2010; Rueda-Orozco et al., 2010; Pava et al., 2014, 2016). In this regard, an experimental approach to explore the role of FAAH in sleep modulation has consisted in the characterization of the properties of FAAH inhibitors, such as URB597 (Murillo-Rodríguez et al., 2007b, 2011a, 2016). Among FAAH inhibitors, several compounds such as 1-heteroarylpropan-2-ones, piperidinyl thiazole isoxazoline, PF-04457845, JNJ-42165279, BIA 10-2474, AM3506, URB694, ARN146333-carboxamido-5-aryl-isoxazole, *N*-aryl 2-aryloxyacetamides have been reported with significant activity. It is worthy to mention that URB597 has been described as one of the most potent FAAH inhibitors so far (Carnevali et al., 2015; Keith et al., 2015; Lodola et al., 2015; Doenni et al., 2016; Panlilio et al., 2016; Pember et al., 2016; Tong et al., 2016; Tuo et al., 2016; Zahov et al., 2017; Sunduru et al., 2017; Wilkerson et al., 2017). However, full description of these FAAH inhibitors in sleep control requires attention.

**FIGURE 13:**
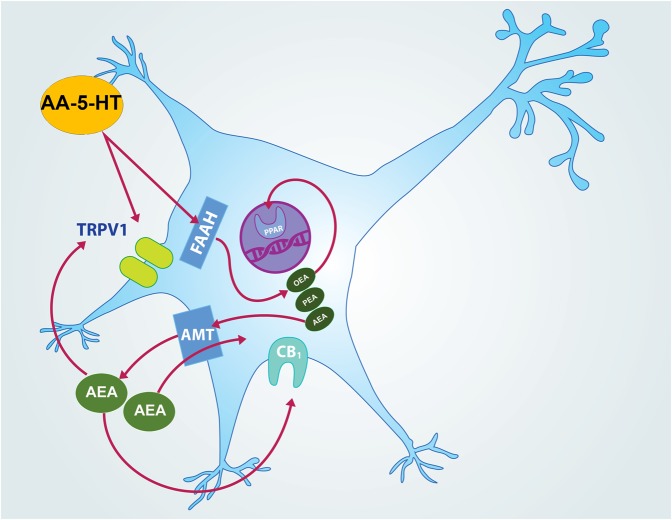
**Hypothetical molecular mechanism of action of AA-5-HT in sleep promotion**. In this empirical proposal, AA-5-HT inhibits fatty acid amide hydrolase (FAAH) which in turn increases levels of oleoylethanolamide (OEA) and palmitoylethanolamide (PEA) as well as anandamide (AEA). Whereas OEA binds to PPARα in nucleus and promotes waking, AEA is able to diffuse across the cellular membrane by anandamide membrane transporter (AMT) to bind either CB_1_ cannabinoid receptor or TRPV1. AA-5-HT can also antagonize AEA action at TRPV1. While activation of CB_1_ cannabinoid receptor has been linked with sleep generation, further evidence is needed to determine whether activation or inhibition of TRPV1 would modulate sleep.

AA-5-HT is a dual blocker of FAAH/TRPV1 that shows multiple pharmacological effects. For instance, this compound produces anxiolytic-like effects in mice tested in the open arms (Micale et al., 2009; John and Currie, 2012). Moreover, AA-5-HT also reduces depression-like behaviors without changing locomotor activity (Navarria et al., 2014; Sartim et al., 2017). It is worthy to note that to our knowledge, the current report provides for the very first time direct evidence about the effects of AA-5-HT in sleep modulation. In this regard, the systemic injection of this drug increased sleep during the lights-off, but not in the lights-on period. Moreover, we found that AA-5-HT enhanced power spectra linked to sleep such as delta and theta whereas neurotransmitters associated to waking were found decreased during the lights-off period. Furthermore, in our experiments, AA-5-HT prevented the blockade of the effects of CBD or MOD on sleep rebound as well as neurotransmitters changes after TSD. Taking together, these data suggest that either FAAH or TRPV1 or both might be modulating sleep, sleep homeostasis and neurotransmitter contents in the presence of wake-promoting compounds such as CBD or MOD. Although a mechanistic approach was not addressed in the current report, we would like to draw the following hypothetical scenario (**Figure 13**): AA-5-HT inhibits FAAH leading to the enhancement in contents of oleoylethanolamide (OEA), palmitoylethanolamide (PEA) as well as AEA (de Lago et al., 2005; Palazzo et al., 2010). Whereas OEA binds to PPARα in the nucleus and promotes waking (Mijangos-Moreno et al., 2016), AEA is able to diffuse across the cellular membrane by AMT to bind either CB_1_ cannabinoid receptors or TRPV1 (Leung et al., 2013; Nicolussi and Gertsch, 2015). While activation of CB_1_ cannabinoid receptor has been linked with sleep generation (Murillo-Rodríguez et al., 2001, 2003, 2016), further evidence is required to fully determine whether activation of TRPV1 modulates sleep. As mentioned previously, FAAH inhibition may also lead to the increase in the levels of other FAAH substrates. Indeed, Wollank et al. (2015) demonstrated higher levels of AEA, 2-AG, OEA, and PEA in cells incubated with AA-5-HT. These data were confirmed by LC-MS analyses in *in vitro* studies showing an enhancement of the levels of AEA-like lipids, including OEA and PEA with URB597 (Winkler et al., 2016). If FAAH inhibition promotes waking (Murillo-Rodríguez et al., 2007b, 2011a), how can we reconcile the current results with available evidence in the literature? Since AEA modulates multiple neurobiological functions also acting via TRPV1 (Di Marzo and De Petrocellis, 2012; Starowicz et al., 2012), then a hypothetical explanation would be that AA-5-HT increases sleep via the involvement of TRPV1 activated by AEA. However, the TRPV1 agonist capsaicin increases the firing rate of rat DA neurons (Marinelli et al., 2005; Chakraborty et al., 2016). Moreover, AA-5-HT promotes the electrophysiological activity of locus coeruleus neurons (de Novellis et al., 2008). It is widely accepted that both brain areas are related with waking since their firing rates are higher across alertness rather than during sleep (Berridge et al., 2012). Thus, discrepancies between current results and available evidence might involve –besides methodological differences between comparable studies- a more complex mechanism of action. It can be proposed that AA-5-HT induces sleep not only by facilitating endogenous AEA action at CB1 cannabinoid receptors but also by antagonizing its activation of TRPV1. Thus our data lead us to speculate that AA-5-HT acts as a dual FAAH inhibitor and TRPV1 antagonist, but this should not necessarily be viewed as predictable, because: (1) the activity of AA-5-HT is exerted at higher concentrations comparted to other more potent FAAH inhibitors, such as URB597, and (2) the role of AEA as a full or partial agonist of TRPV1 to promote sleep lacks full investigation. Nevertheless, AA-5-HT could show higher preference for binding to TRPV1 resulting in an increase in AEA levels which in turn will lead to sleep promotion via activation of CB_1_ cannabinoid receptors and not to sleep inhibition via TRPV1. Studies addressing the displacement of AA-5-HT from binding sites of TRPV1 simultaneously with AEA will provide new insights regarding endocannabinoid system function.

Lastly, although there are no comparative studies between the different experimental approaches on what could be the most reliable strategy for the manipulation of the endocannabinoid system (endogenous ligands, receptors, transporters, synthesizing/degrading enzymes, etc.) in sleep modulation, the current report provides further evidence for the role of AA-5-HT in sleep control. At this date, the neuromolecular role of the endocannabinoid system in sleep-wake cycle regulation has provided important insights regarding the involvement of AEA, CB_1_ cannabinoid receptor, AMT, as well as FAAH (Murillo-Rodríguez et al., 1998, 2001, 2003, 2007b, 2008a, 2011a, 2016; Herrera-Solis et al., 2010; Pava et al., 2014, 2016; Rueda-Orozco et al., 2010). However, the identification of new compounds targeting the endocananbinoid system will help validating the role of this system in sleep control highlighting the most pertinent drug with highest efficiency and lowest side effects in animal models (Lauria et al., 2015; Nimczick and Decker, 2015; Bertini et al., 2016; Aizpurua-Olaizola et al., 2017; Chicca et al., 2017).

Indeed, studies are needed to fully describe the mechanism of action by which AA-5-HT promotes sleep and modulates power spectra activity. Moreover, the regulation of enzymes responsible for neurotransmitter synthesis and/or degradation under the influence of AA-5-HT is also unknown. In addition, the role of AA-5-HT as a blocker of alertness caused by CBD or MOD during the sleep rebound period after TSD still requires further studies.

## Conclusion

In summary, AA-5-HT promotes sleep and decreases waking-related neurotransmitters in the lights-off period, and modulates sleep homeostasis in the presence of wake-promoting compounds such as CBD or MOD. The full understanding of the mechanisms through which AA-5-HT modulates sleep awaits further investigation.

## Author Contributions

EM-R designed, performed the experiments, analyzed data and wrote the Manuscript. VD provided methodological support and contributed to writing the manuscript. SM revised experimental design. NBR, ABV, GAMN, HB, OA-C, revised analyzed data. GA-S provided methodological support. All authors revised the whole paper and approved the final version of the manuscript.

## Conflict of Interest Statement

The authors declare that the research was conducted in the absence of any commercial or financial relationships that could be construed as a potential conflict of interest.
